# Auxiliary-Cavity-Assisted Slow and Fast Light in a Photonic Molecule Spinning Optomechanical System

**DOI:** 10.3390/mi14030655

**Published:** 2023-03-14

**Authors:** Hua-Jun Chen, Yun-He Liu, Bao-Hao Xie

**Affiliations:** School of Mechanics and Photoelectric Physics, Anhui University of Science and Technology, Huainan 232001, China

**Keywords:** spinning resonator, optomechanically induced transparency, slow light, coherent light propagation

## Abstract

We investigate the coherent optical propagation in a photonic molecule spinning optomechanical system consisting of two whispering gallery microcavities in which one of the optical cavities is a spinning optomechanical cavity and the other one is an ordinary auxiliary optical cavity. As the optomechanical cavity is spinning along the clockwise or counterclockwise direction, the cavity field can undergo different Sagnac effects, which accompanies the auxiliary optical cavity, together influencing the process of the evolution of optomechanically induced transparency and its related propagation properties, such as fast and slow light effects. The numerical results indicate that the enhanced slow and fast light and the conversion from fast to slow light (or slow to fast light) are determined by the spinning direction of the optomechanical cavity and the coupling of the two optical cavities. The study affords further insight into the photonic molecule spinning optomechanical systems and also indicates promising applications in quantum information processing.

## 1. Introduction

Cavity optomechanical systems [1,2] have witnessed significant progress in the past few decades, both in potential applications, including ultra-high-precision detection [3,4,5,6,7] and quantum information processing [8,9,10,11], and also as ideal systems for cavity QED experiments [1]. The optomechanical phenomena come from the radiation pressure forces inducing phonon modes, which in turn influence the cavity optical properties, as a result leading to distinct quantum interference effects. Then, a good deal of breakthroughs, including squeezing [12,13], phonon lasers [14,15,16], nonreciprocity [17], entanglement [10,11], and exceptional points [16,18,19,20], have been demonstrated in different types of cavity optomechanical systems. Specifically, another interesting phenomenon related to the present work is optomechanically induced transparency (OMIT), which was also demonstrated in different optomechanical systems [13,21,22,23,24,25]. OMIT arises from the destructive interference of two absorption channels of the probe photons and manifests significant applications in slow light [22,26,27], sensing [28,29,30,31], information storage [32,33], and so on.

Recently, a spinning optical cavity, i.e., a whispering gallery mode (WGM) optical cavity in the optomechanical system is rotating [34], has drawn widespread attention, and the spinning resonator has been demonstrated in a very recent experiment [35]. When the WGM resonator is spinning, the clockwise (CW) and counterclockwise (CCW) optical modes in the WGM cavity will undergo different Sagnac–Fizeau shifts. Based on the spinning resonator systems and Sagnac–Fizeau effect, many phenomena and applications, such as phonon lasers [36], nanoparticle sensing [37], the nonreciprocal photon blockade effect [38,39], breaking anti-PT symmetry [40], and entanglement generation [41], have been demonstrated.

In the present article, based on the spinning WGM resonator [34,35], we further introduce another ordinary WGM optical cavity to form the photonic molecule spinning optomechanical system. We aim to investigate the optical responses of the spinning WGM resonator system with an auxiliary optical cavity, where the WGM resonator rotating along the CW and CCW direction will experience different Sagnac–Fizeau shifts, and the spinning directions accompanied by the auxiliary optical cavity and mechanical mode together influence the probe field transmission. Controlling the system parameters of the cavity–cavity coupling and the direction of rotation of the WGM cavity, OMIT in the probe transmission spectra vary significantly. Finally, we demonstrate that the slow light is affected by numerically calculating the group delay of the probe field around the transparency window accompanied by the steep phase dispersion under different parametric regimes, and the results indicate that the tunable slow and fast light effect can be easily obtained by modulating several system parameters.

## 2. Model and Theory

**Figure 1:**
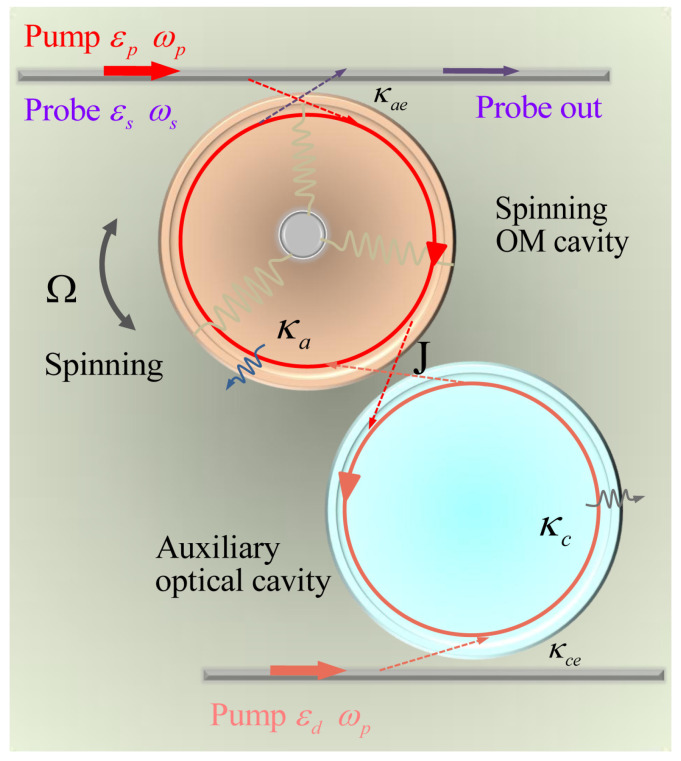
Schematic diagram of the photonic molecule spinning optomechanical system, which includes a spinning optomechanical cavity and one ordinary auxiliary optical cavity. The optomechanical cavity is driven by a pump field and a probe field, while the auxiliary optical cavity is only driven by a pump field. The spinning optomechanical cavity can spin along the CW and CCW direction, and *J* indicates the coupling of the two cavities.

The model to be studied is shown in Figure 1, where a rotating WGM resonator **a** with resonance frequency $\omega_{a}$ and intrinsic loss rate $\kappa_{a}$ is evanescently coupled to a tapered fiber. The cavity **a** is driven by a strong pump field with frequency $\omega_{p}$ and a weak probe field with frequency $\omega_{s}$ where the amplitude of the pump field (probe field) is $\varepsilon_{p}=\sqrt{P_{c}/\hbar \omega_{p}}$ ($\varepsilon_{s}=\sqrt{P_{s}/\hbar \omega_{s}}$), where $P_{c}$ ($P_{s}$) is the pump (probe) field power. Due to the radiation pressure, the resonator supports a mechanical breathing mode with the frequency $\omega_{m}$. When the WGM resonator **a** is spinning along the CW or CCW direction with an angular velocity Ω, the light circulating in the cavity **a** experiences a Sagnac–Fizeau shift [42,43,44], i.e., $\omega_{a}\to \omega_{a}+\Delta_{SF}$, with $\Delta_{SF}=\pm \frac{nR\Omega \omega_{a}}{c}\left(1-\frac{1}{n^{2}}-\frac{\lambda }{n}\frac{dn}{d\lambda }\right)\equiv \pm \eta \Omega$, where *n* is the refractive index, *R* is the radius of the WGM resonator, and *c* (λ) is the speed (wavelength) of light in a vacuum. Generally, the dispersion term dn/dλ describing the relativistic component is quite small [35,43]. The cavity **c** is an ordinary WGM optical cavity with resonance frequency $\omega_{c}$ and intrinsic loss rate $\kappa_{c}$, which is driven by pump field $\varepsilon_{d}=\sqrt{P_{d}/\hbar \omega_{p}}$ with $P_{d}$ as the pump field power.

In the frame rotation of the pump field, the Hamiltonian of the hybrid system is [34,45,46,47]

(1) $$\begin{array}{ccc} H & = & \hbar \Delta a^{†}a+\hbar \Delta_{c}c^{†}c+\left(\frac{p^{2}}{2M}+\frac{1}{2}M\omega_{m}^{2}x^{2}\right)+\frac{p_{\theta }^{2}}{2MR^{2}}-\hbar ga^{†}ax+\hbar J\left(a^{†}c+ac^{†}\right)\\ \; & \; & +i\hbar \sqrt{\kappa_{ae}}\varepsilon_{p}\left(a^{†}-a\right)+i\hbar \sqrt{\kappa_{ae}}\varepsilon_{s}\left(a^{†}e^{-i\delta t}-ae^{i\delta t}\right)+i\hbar \sqrt{\kappa_{ce}}\varepsilon_{d}\left(c^{†}-c\right), \end{array}$$

where $\Delta =\Delta_{a}\pm \Delta_{SF}=\omega_{a}-\omega_{p}\pm \Delta_{SF}$ is the detuning of the cavity **a** and the pump field. If the WGM rotates along the CW direction, $\Delta =\Delta_{a}+\Delta_{SF}$; and in the CCW direction, $\Delta =\Delta_{a}-\Delta_{SF}$. $a^{†}\left(a\right)$ is the creation (annihilation) operator of the optical cavity mode **a**, $c^{†}\left(c\right)$ is the creation (annihilation) operator of the optical cavity mode **c**, and *x* and *p* are the displacement and momentum operators of the phonon mode with the commutation relation $\left[x,p\right]=i$. $g=\omega_{c}/R$ is the optomechanical coupling strength; θ and $p_{\theta }$ denote the rotation angle and angular momentum operators with the commutation relation $\left[\theta ,p_{\theta }\right]=i$ [47]. $\kappa_{ae}$ and $\kappa_{ce}$ are the extra losses of cavity **a** and cavity **c**, and we consider $\kappa_{ae}=\kappa_{a0}$ and $\kappa_{ce}=\kappa_{c0}$, i.e., the optical loss of the cavities κ can be denoted as $\kappa_{a}=\kappa_{ae}+\kappa_{a0}$ and $\kappa_{c}=\kappa_{ce}+\kappa_{c0}$. $\delta =\omega_{s}-\omega_{p}$ is the detuning of the probe field and pump field.

We then can obtain the quantum Langevin equations (QLEs) of the system as follows:

(2) $$\dot{a}=-\left(i\Delta +\kappa_{a}\right)a+igxa-iJc+\sqrt{\kappa_{ae}}\varepsilon_{p}+\sqrt{\kappa_{ae}}\varepsilon_{s}e^{-i\delta t},$$

(3) $$\dot{c}=-\left(i\Delta_{c}+\kappa_{c}\right)c+iJa+\sqrt{\kappa_{ce}}\varepsilon_{d},$$

(4) $$\ddot{x}+\gamma_{m}\dot{x}+\omega_{m}^{2}x=\frac{\hbar g}{M}c^{†}c+\frac{p_{\theta }^{2}}{M^{2}R^{3}},$$

(5) $$\dot{\theta }=\frac{p_{\theta }}{MR^{2}},\;\;\;\;\;{\dot{p}}_{\theta }=0,\;$$

where $\gamma_{m}$ is the mechanical mode damping rate and *M* is the effective mass of the resonator. Due to the strong optical pump, we can linearize the dynamics by expanding each operator as a sum of its steady-state value and a small fluctuation around it, i.e., $\rho =\rho_{s}+\delta \rho$ (ρ indicates the operators: *a*, *c*, *x*), and we can obtain three steady-state equations as $\left(i\Delta {}^{\prime }+\kappa_{a}\right)a_{s}+iJc_{s}=\sqrt{\kappa_{ae}}\varepsilon_{p}$, $\left(i\Delta_{c}+\kappa_{c}\right)c_{s}+iJa_{s}=\sqrt{\kappa_{ce}}\varepsilon_{d}$, and $x_{s}=\frac{\hbar g}{M\omega_{m}^{2}}{\left|c_{s}\right|}^{2}+R{\left(\frac{\Omega }{\omega_{m}}\right)}^{2}$ with $\Delta {}^{\prime }=\Delta -gx_{s}$, which together determine the intracavity photon number ${\left|a_{s}\right|}^{2}$ and ${\left|c_{s}\right|}^{2}$.

Considering that the pump field is sufficiently strong, all the operators can be identified with their expectation values using the mean-field approximation $\langle Qc\rangle =\langle Q\rangle \langle c\rangle$ [13]. After being linearized by neglecting nonlinear terms in the fluctuations, the QLEs for the expectation values are as follows:

(6) $$\langle \delta \dot{a}\rangle =-\left(i\Delta {}^{\prime }+\kappa_{a}\right)\langle \delta a\rangle +iga_{s}\langle \delta x\rangle -iJ\langle \delta c\rangle +\sqrt{\kappa_{ae}}\varepsilon_{s}e^{-i\delta t},$$

(7) $$\langle \delta \dot{c}\rangle =-\left(i\Delta_{c}+\kappa_{c}\right)\langle \delta c\rangle -iJ\langle \delta a\rangle ,$$

(8) $$\langle \delta \ddot{x}\rangle +\gamma_{m}\langle \delta \dot{x}\rangle +\omega_{m}^{2}\langle \delta x\rangle =\frac{\hbar g}{M}\left(a_{s}^{*}\langle \delta a\rangle +a_{s}\langle \delta a^{†}\rangle \right).$$

To solve Equations (6)–(8), we obtain the ansatz [48] as $\langle \delta \rho \rangle =\rho_{+}e^{-i\delta t}+\rho_{-}e^{i\delta t}$, substituting them into the above equations while ignoring the high-order terms and working to the lowest order in $\varepsilon_{s}$ but to all orders in $\varepsilon_{p}$; then, we obtain

(9) $$a_{+}=\frac{\left(\kappa_{a}-i\Lambda_{2}\right)\sqrt{\kappa_{ae}}\varepsilon_{s}}{\left(\kappa_{a}+i\Lambda_{1}\right)\left(\kappa_{a}-i\Lambda_{2}\right)-\hbar^{2}g^{4}\chi^{2}{\left|c_{s}\right|}^{4}},$$

where $\chi =1/M(-\delta^{2}-i\delta \gamma_{m}+\omega_{m}^{2})$, $\Lambda_{1}=\Delta {}^{\prime }-\delta -\hbar g^{2}\chi {\left|a_{s}\right|}^{2}+J\theta_{1}$, $\Lambda_{2}=\Delta {}^{\prime }+\delta -\hbar g^{2}\chi {\left|a_{s}\right|}^{2})+J\theta_{2}$, $\theta_{1}=-iJ/\left[i\left(\Delta_{c}-\delta \right)+\kappa_{c}\right]$, and $\theta_{2}=-iJ/\left[-i\left(\Delta_{c}+\delta \right)+\kappa_{c}\right]$. Using the standard input–output relation [49] $a_{out}\left(t\right)=a_{in}\left(t\right)-\sqrt{2\kappa_{a}}a\left(t\right)$, where $a_{out}\left(t\right)$ is the output field operator, the transmission rate of the probe field is [21,22,23,50]

(10) $$T={\left|t(\omega_{s})\right|}^{2}={\left|\frac{a_{out}\left(t\right)}{a_{in}\left(t\right)}\right|}^{2}=\left|1-\frac{\sqrt{\kappa_{ae}}a_{+}}{\varepsilon_{s}}\right|.$$

In order to investigate the group delay, we introduce group delay $\tau_{g}$, which is defined by

(11) $$\tau_{g}=\frac{d\phi_{t}}{d\omega_{s}}=\frac{d\{\arg\left[t\left(\omega_{s}\right)\right]\}}{d\omega_{s}},$$

where $\phi_{t}=\arg\left[t\left(\omega_{s}\right)\right]$ is the phase dispersion. Theoretical research has demonstrated that a positive group delay (i.e., $\tau_{g}>0$) indicates slow light, while the negative delay group (i.e., $\tau_{g}<0$) refers to fast light, respectively.

## 3. Numerical Results and Discussion

**Figure 2:**
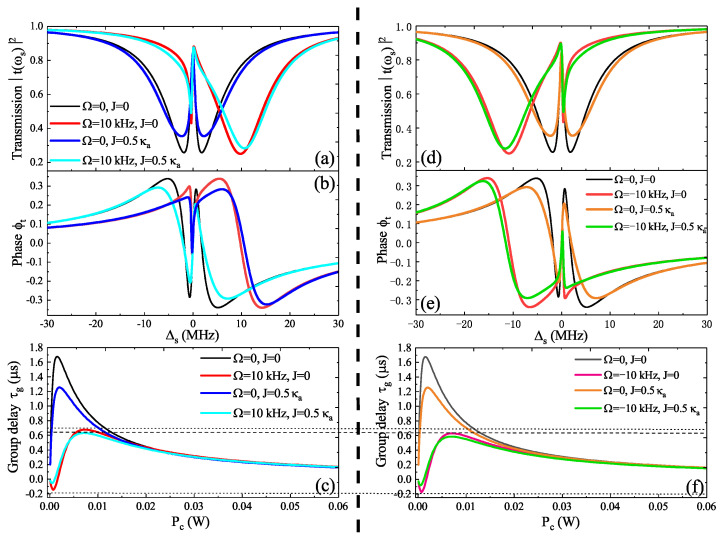
(**a**,**b**) The transmission and phase $\phi_{t}$ versus $\Delta_{s}$ for different parameter regimes under the condition of Ω>0. (**c**) The group delay $\tau_{g}$ versus $P_{c}$ for different parameter regimes corresponding to (**a**,**b**). (**d**,**e**) The probe transmission and the phase $\phi_{t}$ versus $\Delta_{s}$ for different parameter regimes in the case of Ω<0. (**f**) The group delay $\tau_{g}$ versus $P_{c}$ for several different parameters corresponding to (**d**,**e**).

Here, we reference the experimentally feasible parameters [51]: the effective mass of the resonator M=20 ng, the frequency of the resonator $\omega_{m}=200$ MHz, the mechanical damping rate $\gamma_{m}=0.2$ MHz, the wavelength of light λ=1.55 μm, the refractive index n=1.44, the speed of light in a vacuum $c=3\times 10^{8}$ m/s, the resonance frequency of WMG **a** $\omega_{a}=193.5$ THz, the quality (Q) factor of optomechanical cavity **c** $Q=\omega_{a}/\kappa_{a}=3\times 10^{7}$, R=0.25 mm, the pump power of cavity **a** $P_{c}=0.01$ W, the pump power of cavity **c** $P_{d}=0.001$ W, and $\Delta_{a}=\Delta_{c}=\omega_{m}$. In Figure 2a,b, we give the transmission $T(\omega_{s})$ and the phase $\phi_{t}$ of the probe light as a function of probe-cavity detuning $\Delta_{s}$ for different parameter regimes, i.e., (Ω=0, J=0), (Ω=10 kHz, J=0), (Ω=0, J=0.5 $\kappa_{a}$), and (Ω=10 kHz, J=0.5 $\kappa_{a}$), respectively. In the case of Ω=0 and J=0, the WGM resonator **a** is stationary and without considering the auxiliary optical cavity **c**, which is a familiar case, and the transmission of the probe field shows the well-known phenomenon of OMIT, which displays symmetrical mode splitting. Then, a transmission window appears around $\Delta_{s}=0$ due to the destructive interference, which has been demonstrated in the WGM optomechanical system [21]. If Ω≠0 (such as Ω=10 kHz, i.e., the WGM resonator **a** rotates along the CW direction), the OMIT phenomenon in the transmission spectrum will be right-shifted, which has been demonstrated in a spinning resonator system [34,52,53]. If we consider that the WGM resonator **a** is stationary but introduce an auxiliary optical cavity **c**, i.e., Ω=0 and J=0.5 $\kappa_{a}$, the symmetrical mode splitting in the transmission spectrum is enhanced as several photons will flow into the WGM resonator **a** from the auxiliary cavity **c**, which was also demonstrated in the photonic molecule optomechanical system [54]. However, we are more concerned with the case of Ω=10 kHz and J=0.5 $\kappa_{a}$, i.e., not only the WGM resonator **a** is rotating but also introducing an auxiliary optical cavity **c**; then, the Sagnac–Fizeau effect and the cavity–cavity coupling *J* will together influence the OMIT phenomenon. The numerical results show that not only the OMIT varies observably but also the unsymmetrical mode splitting is enhanced, which accompanies the phase around $\Delta_{s}=0$ changing significantly. Figure 2c gives the group delay $\tau_{g}$ as a function of pump power $P_{c}$ for four different parameter regimes corresponding to Figure 2a,b. In the parameter regimes of (Ω=0, J=0) and (Ω=0, J=0.5 $\kappa_{a}$), the group delay $\tau_{g}$ indicates a slow light effect, where $\tau_{g}$ first reaches a maximum value and then reduces gradually and finally reaches stabilization with increasing pump power $P_{c}$. Meanwhile, in the parameter regimes of (Ω=10 kHz, J=0) and (Ω=10 kHz, J=0.5 $\kappa_{a}$), with increasing pump power $P_{c}$, the group delay $\tau_{g}$ first reaches a minimum value and then reaches a maximum and finally reduces gradually to stabilization, where $\tau_{g}$ experiences conversion from negative to positive, corresponding to the the conversion from a fast light to a slow light effect. Therefore, we can conclude that the auxiliary optical cavity **c** leads to slow light and the rotation of the WGM resonator **a** results in the transition from fast to slow light.

In Figure 2d,e, we display the transmission $T(\omega_{s})$ and the phase $\phi_{t}$ of the probe light versus the detuning $\Delta_{s}$ for different parameter regimes, i.e., (Ω=0, J=0), (Ω= −10 kHz, J=0), (Ω=0, J=0.5 $\kappa_{a}$), and (Ω= −10 kHz, J=0.5 $\kappa_{a}$), respectively. Compared with Figure 2a, the difference in Figure 2d is that the WGM resonator **a** rotates along the CCW direction, and, consequently, the OMIT spectra in the transmission spectrum will be left-shifted, as shown in Figure 2d. Figure 2f also gives the group delay $\tau_{g}$ versus the pump power $P_{c}$ for four different parameter regimes corresponding to Figure 2d,e; the auxiliary optical cavity **c** also leads to the slow light effect and the rotation of the WGM resonator **a** results in the transition from fast to slow light. However, compared with Figure 2c,f, we can find that the WGM resonator **a** rotating along the CW direction (i.e., Ω>0) will enhance the slow light, while, if the WGM resonator **a** is rotating along the CCW direction (i.e., Ω<0), the fast light effect will be enhanced. Therefore, in the following, we will extensively investigate the Sagnac–Fizeau effect and the cavity–cavity coupling *J* that together influence the OMIT phenomenon and the coherent optical propagation under different parameter regimes.

**Figure 3:**
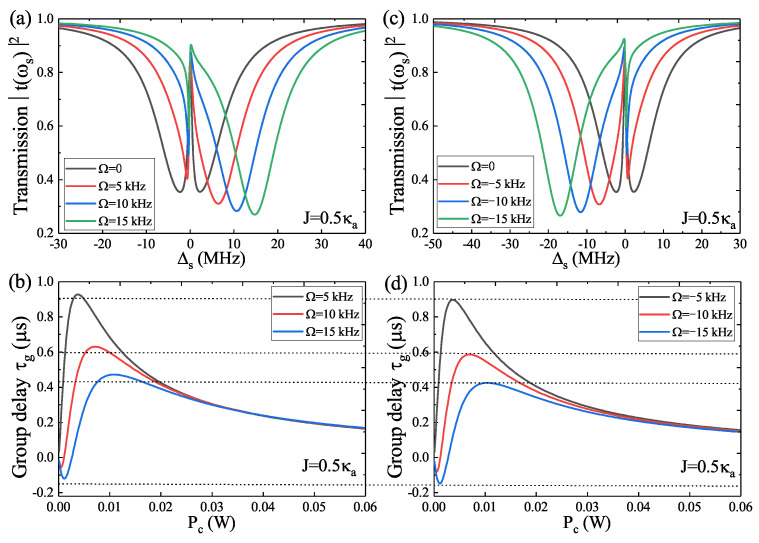
(**a**,**b**) The transmission $T(\omega_{s})$ and the group delay $\tau_{g}$ for different angular velocities Ω rotating along the CW direction (Ω>0) at J=0.5 $\kappa_{a}$. (**c**,**d**) The transmission $T(\omega_{s})$ and the group delay $\tau_{g}$ for different angular velocities Ω rotating along the CCW direction (Ω<0) at J=0.5 $\kappa_{a}$.

In Figure 3, we introduce an auxiliary optical cavity **c** into the spinning optomechanical system to form the photonic molecule spinning optomechanical system, where, once the rotation of the WGM resonator **a** is taken into consideration, both the OMIT and the fast–slow light will be varied significantly. In Figure 3a, we present the transmission $T(\omega_{s})$ versus $\Delta_{s}$ at fixed cavity–cavity coupling J=0.5 $\kappa_{a}$ for four different angular velocities Ω rotating along the CW direction. When increasing the parameter Ω from Ω=0 to Ω=15 kHz, the transmission spectra experience a conversion from symmetrical splitting to unsymmetrical splitting, accompanying a right-shifted peak and increased peak separation. Figure 3b gives the group delay $\tau_{g}$ as a function of the pump power $P_{c}$ for three velocities Ω rotating along the CW direction, and we can see that if the rotational velocity of the WGM resonator **a** is small (such as Ω=5 kHz), the group delay $\tau_{g}$ indicates that the slow light is dominant (i.e., $\tau_{g}>0$), while, if we further increase the rotational velocities Ω (such as Ω⩾10 kHz), the group delay $\tau_{g}$ experiences a transition from negative to positive, i.e., the transition from fast to slow light. Figure 3c plots the transmission $T(\omega_{s})$ versus $\Delta_{s}$ at fixed cavity–cavity coupling J=0.5 $\kappa_{a}$ for four different angular velocities Ω along the CCW direction, and the results are the same as in Figure 3a, while the only difference is that the transmission peaks in Figure 3c shift to the left due to the WGM resonator **a** spinning along the CCW direction. In Figure 3d, we give the group delay $\tau_{g}$ versus $P_{c}$ for three velocities Ω along the CCW direction, and the results are the same as in Figure 3b. However, compared with the group delay $\tau_{g}$ in Figure 3b,d, for a fixed cavity–cavity coupling *J*, the slow light effect is greater in the condition of the WGM resonator **a** rotating along the CW direction than in the case of the WGM resonator rotating along the CCW direction. Meanwhile, for the fast light effect, the fast light is more remarkable in the condition of Ω<0 than in the case of Ω>0.

**Figure 4:**
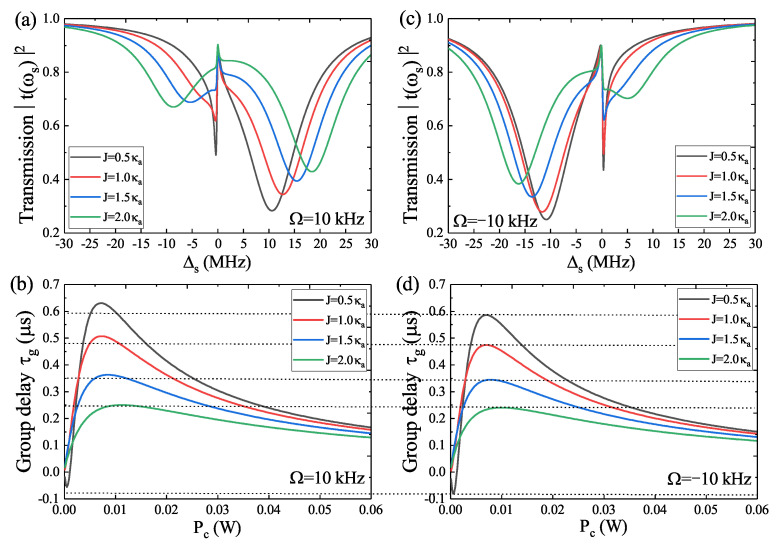
(**a**,**b**) The transmission $T(\omega_{s})$ and the group delay $\tau_{g}$ for different cavity–cavity coupling *J* in condition of Ω=10 kHz. (**c**,**d**) The transmission $T(\omega_{s})$ and the group delay $\tau_{g}$ for different cavity–cavity coupling *J* in condition of Ω= −10 kHz.

On the other hand, the transmission spectra $T(\omega_{s})$ versus $\Delta_{s}$ with increasing cavity–cavity coupling *J* from J=0.5 $\kappa_{a}$ to J=2.0 $\kappa_{a}$ are also investigated under the WGM resonator **a** spinning along the CW (Ω=10 kHz) and CCW (Ω=−10 kHz) direction, respectively, as shown in Figure 4a,c. When the auxiliary optical cavity **c** is taken into consideration, the photons in cavity **c** will flow into WGM resonator **a**, which results in broader transmission spectra. Accompanying the WGM resonator **a** spinning along the CW and CCW direction, the transmission spectra present a right shift and left shift, respectively. In Figure 4b,d, we plot the group delay $\tau_{g}$ for several different values of cavity–cavity coupling *J* under the resonator **a** spinning along (Ω=10 kHz) and CCW (Ω= −10 kHz) directions, respectively. Obviously, for the unchanged angular velocities Ω=10 kHz and Ω= −10 kHz, in the case of J=0.5 $\kappa_{a}$, the group delay $\tau_{g}$ first reduces to a minimum value and then continuously increases until reaching a maximum value and finally decreases to a saturated value. Thus, the rotation along the CW direction of the resonator **a** induces greater slow light and the CCW rotation of the resonator **a** leads to an enhanced fast light effect. When J⩾1.0 $\kappa_{a}$, the group delay $\tau_{g}$ only manifests the slow light, and the slow light effect is larger in the situation of the resonator **a** rotating in the CW direction than in the condition of the CCW direction.

**Figure 5:**
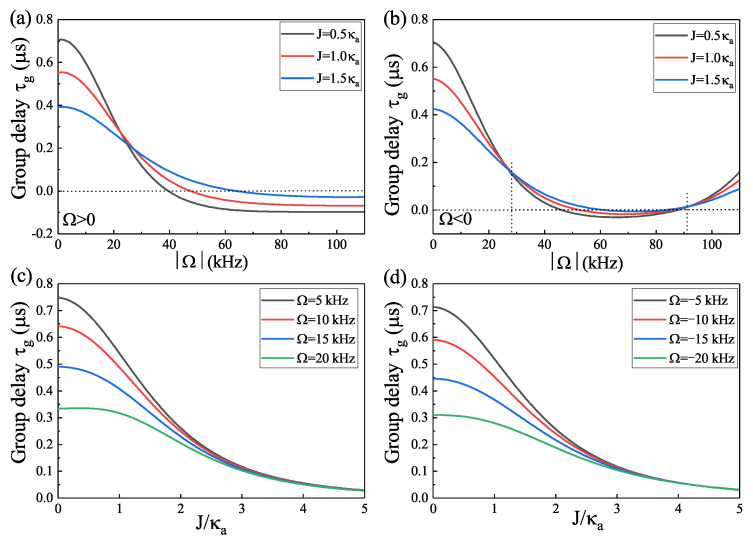
(**a**) The group delay varying with $\left|\Omega \right|$ in the case of Ω>0 for three *J*. (**b**) The group delay varying with $\left|\Omega \right|$ in the case of Ω<0 for three *J*. (**c**) The group delay varying with *J* for different angular velocities Ω along the CW direction (Ω>0). (**d**) The group delay varying with *J* for different angular velocities Ω spinning along the CCW direction (Ω<0).

Furthermore, the group delay $\tau_{g}$ varying with the rotational velocity $\left|\Omega \right|$ spinning along the CW direction (Ω>0) for three different values of cavity–cavity coupling *J* is shown in Figure 5a, and the results manifest that $\tau_{g}$ continuously reduces from a maximum value and then reaches a saturated numerical value. The process indicates that $\tau_{g}$ experiences a conversion from slow to fast light. When the resonator **a** rotates along the CCW direction (i.e., Ω<0), the group delay $\tau_{g}$ continuously reduces from a maximum value and then reaches a saturated numerical value, and if we further increase rotational velocity $\left|\Omega \right|$ along CCW, the group delay $\tau_{g}$ begins to increase and reaches a submaximal value, as shown in Figure 5b. The results in Figure 5b manifest that the group delay $\tau_{g}$ experiences a transition of slow–fast–slow light. In Figure 5c,d, we consider the rotational angular velocities Ω rotating along the CW and CCW direction, respectively, and investigate the group delay $\tau_{g}$ varying with the cavity–cavity coupling *J* for different Ω. It is obvious that the group delay $\tau_{g}$ indicates slow light and continuously reduces from a maximum value and then reaches a saturated numerical value for a fixed Ω. Compared with the slow light effect in Figure 5c,d, for a fixed rotational angular velocity Ω, the slow light effect is more remarkable in the situation of the WGM resonator **a** spinning along the CW direction than in the circumstance of rotation along the CCW direction. Therefore, not only the spinning direction of the resonator **a** but also the cavity–cavity coupling *J* together influence the slow light effect.

Finally, according to the numerical results from Figure 2, Figure 3, Figure 4 and Figure 5, we can obtain the following conclusions: (1) the spinning direction of the WGM resonator **a** and the cavity–cavity coupling *J* together determine the process of evolution of the transmission spectra; (2) if the WGM resonator **a** spins along the CW direction, the slow light effect is enhanced, while, if the WGM resonator **a** spins along the CCW direction, the fast light effect is more distinct; (3) the parameters of the cavity–cavity coupling *J*, the Sagnac–Fizeau shift $\Delta_{SF}$, and the spinning direction (Ω<0 or Ω>0) together determine the coherent optical propagation properties.

## 4. Conclusions

In conclusion, we have demonstrated the optical response properties in the photonic molecule spinning optomechanical cavity, where a rotating WGM optomechanical cavity is coupled to another ordinary optical cavity. When the optomechanical cavity rotates along the CW and CCW direction, the cavity will undergo different Sagnac frequency shifts, which strongly influences the transmission and the group delay of the probe field; accompanying the role of the auxiliary optical cavity, OMIT in the transmission spectra and its related optical propagation properties vary significantly. The results indicate that the optomechanical cavity rotating along the CW direction results in an enhanced slow light effect, while, if the optomechanical cavity rotates along the CCW direction, the fast light effect is deepened. In addition, not only the spinning direction of the optomechanical cavity but also the auxiliary optical cavity together determine the transition from the slow light to the fast light effect.

## Data Availability

Data presented in this article are available on request from the corresponding author.
